# Erythropoietin drives breast cancer progression by activation of its receptor EPOR

**DOI:** 10.18632/oncotarget.16368

**Published:** 2017-03-18

**Authors:** Ka Kui Chan, Kyle B. Matchett, Jonathan A. Coulter, Hiu-Fung Yuen, Cian M. McCrudden, Shu-Dong Zhang, Gareth W. Irwin, Matthew A. Davidson, Thomas Rülicke, Sophie Schober, Ludger Hengst, Heidelinde Jaekel, Angela Platt-Higgins, Philip S. Rudland, Ken I. Mills, Perry Maxwell, Mohamed El-Tanani, Terence R. Lappin

**Affiliations:** ^1^ Centre for Cancer Research and Cell Biology, Queen's University Belfast, Belfast BT9 7AE, UK; ^2^ School of Pharmacy, Queen's University Belfast, Belfast BT9 7AE, UK; ^3^ Institute of Laboratory Animal Science, University of Veterinary Medicine Vienna, Vienna A-1210, Austria; ^4^ Division of Medical Biochemistry, Biocenter, Innsbruck Medical University, Innsbruck A-6020, Austria; ^5^ Institute of Integrative Biology, University of Liverpool, Liverpool L69 3BX, UK; ^6^ Northern Ireland Molecular Pathology Laboratory, Belfast Health & Social Care Trust, Queen's University Belfast, Belfast BT9 7AE, UK; ^7^ Department of Pathology, The University of Hong Kong, Hong Kong Special Administrative Region Hong Kong 999077, China; ^8^ Northern Ireland Centre for Stratified Medicine, Biomedical Sciences Research Institute, Ulster University, Londonderry BT47 6SB, UK; ^9^ Institute of Cancer Therapeutics, University of Bradford, Bradford, West Yorkshire BD7 1DP, UK

**Keywords:** EPO, EPOR, breast cancer, MYC, apoptosis

## Abstract

Breast cancer is a leading cause of cancer-related deaths. Anemia is common in breast cancer patients and can be treated with blood transfusions or with recombinant erythropoietin (EPO) to stimulate red blood cell production. Clinical studies have indicated decreased survival in some groups of cancer patients treated with EPO. Numerous tumor cells express the EPO receptor (EPOR), posing a risk that EPO treatment would enhance tumor growth, but the mechanisms involved in breast tumor progression are poorly understood.

Here, we have examined the functional role of the EPO-EPOR axis in pre-clinical models of breast cancer. EPO induced the activation of PI3K/AKT and MAPK pathways in human breast cancer cell lines. EPOR knockdown abrogated human tumor cell growth, induced apoptosis through Bim, reduced invasiveness, and caused downregulation of MYC expression. EPO-induced MYC expression is mediated through the PI3K/AKT and MAPK pathways, and overexpression of MYC partially rescued loss of cell proliferation caused by EPOR downregulation. In a xenotransplantation model, designed to simulate recombinant EPO therapy in breast cancer patients, knockdown of EPOR markedly reduced tumor growth.

Thus, our experiments *in vitro* and *in vivo* demonstrate that functional EPOR signaling is essential for the tumor-promoting effects of EPO and underline the importance of the EPO-EPOR axis in breast tumor progression.

## INTRODUCTION

Breast cancer is a leading cause of cancer-related deaths, with 1 in 8 women and 1 in 870 men expected to be diagnosed with the disease during their lifetime. Anemia is an independent prognostic risk factor in cancer patients [1] and is a frequent complication in breast cancer. Although recombinant human EPO is an effective treatment for anemia, adverse reports of decreased survival in some groups of cancer patients in Phase III trials led to a decline in its use from 2007 onwards [2] [3].

EPO regulates the survival, proliferation and differentiation of erythroid progenitor cells through activation of its transmembrane receptor, EPOR [4]. Besides hematopoiesis, *EPO* has pleiotropic roles in a diverse range of tissues [5, 6]. EPO and EPOR expression in neoplasia were first reported in clear cell and chromophilic cell renal carcinoma [7] and subsequently functional autocrine and paracrine EPO-EPOR systems were identified in human breast carcinoma, melanoma, prostate cells, and cervical cancer cells [8] suggesting a link to tumor progression. Although EPOR expression on tumor cells is typically several orders of magnitude lower than on erythroid progenitor cells [9], EPO can still activate cell signaling cascades in tumor cells, such as in differentiated neuroblastoma SH-SY5Y cells, which have fewer than 50 EPOR dimers on their cell surface [10]. The observation that some cells, such as astrocytes, are capable of producing both EPO and EPOR pointed to a functional role for EPO as an endocrine, autocrine and paracrine factor involving multiple organs [11].

Two recent clinical studies implicate EPOR in breast tumor growth. In estrogen receptor-positive/progesterone receptor-positive ER(+)/PR(+) tumors, impaired tamoxifen response was correlated with high EPOR expression [12]. Tamoxifen treatment significantly increased recurrence-free survival in patients with ER(+)/PR(+) tumors with low EPOR expression but had no effect on recurrence-free survival in patients with tumors with high EPOR expression. In contrast, recurrence-free survival was significantly improved in patients with ER(+) tumors with high EPOR expression in the untreated cohort, implying that EPOR expression in breast cancer affects tumor behavior.

In HER2-positive metastatic breast cancer, concurrent administration of recombinant EPO and trastuzumab correlated with shorter progression-free survival and overall survival compared to trastuzumab treatment alone [13]. Moreover, exposure of HER2 and EPOR dual-positive breast cancer cell lines to trastuzumab inhibited AKT and ERK phosphorylation, but the inhibition was reduced by simultaneous treatment with recombinant EPO. Taken together these reports suggest that EPOR expression affects breast tumor progression.

The causative effects of rhEPO and autocrine/paracrine EPO production on tumor progression are poorly understood. Here we have examined the impact of EPOR modulation in breast cancer cell lines and in a xenotransplantation model designed to simulate EPO treatment in cancer patients. A coherent picture has emerged, firmly linking the EPO-EPOR axis to breast cancer progression.

## RESULTS

### EPO induces the activation of PI3K/AKT and MAPK pathways in human cancer cell lines

**Figure 1:**
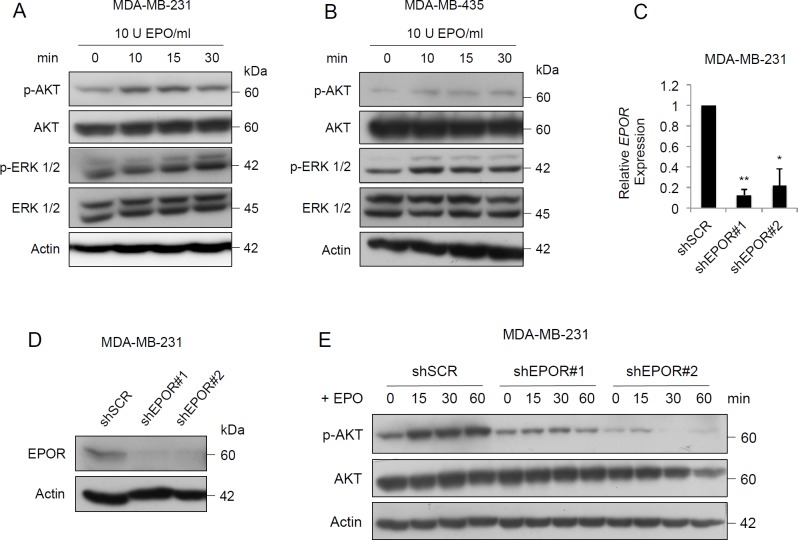
EPO activates PI3K/AKT and MAPK signaling pathways in breast cancer cells **A**. Immunoblot of signaling induced by 10 U EPO/ml in MDA-MB-231 cells and **B**. MDA-MB-435 cells. **C**. *EPOR* mRNA expression in MDA-MB-231-shSCR, MDA-MB-231-shEPOR#1 and MDA-MB-231-shEPOR#2 cells, generated by infection of pLKO.1-scramble (shSCR), pLKO.1-shEPOR#1 (shEPOR#1) and pLKO.1-shEPOR#2 (shEPOR#2), harvested 72 hours after lentiviral transduction. Data shown are means ± SEM. shSCR *vs* shEPOR#1, **p = 0.0037; shSCR *vs* shEPOR#2, *p = 0.0391 by paired t test. **D**. Immunoblot of EPOR in MDA-MB-231-shSCR, MDA-MB-231-shEPOR#1 and MDA-MB-231-shEPOR#2 cells, harvested 72 hours after lentiviral transduction. **E**. Time course analysis of p-AKT and AKT following the addition of 10 U/ml EPO.

In erythroid progenitor cells, EPO binds to EPOR and promotes survival, proliferation and differentiation through three main signaling pathways JAK2/STAT5, PI3K/AKT and MAPK. We investigated the function of EPOR in these signaling pathways in MDA-MB-231 and MDA-MB-435 cells using the clinically relevant concentration of 10 U EPO/ml which activated the PI3K/AKT and MAPK pathways in both cell lines within 10 minutes, as indicated by increased phospho-AKT (pAKT) and phospho-ERK 1/2 (pERK1/2) expression. There were no significant changes in the total AKT or total ERK 1/2 in MDA-MB-231 cells (Figure 1A) or in MDA-MB-435 cells (Figure 1B). EPO had no effect on the JAK2/STAT5 pathway in either cell line (data not shown). To investigate whether activation of the two pathways is mediated specifically by EPOR, we knocked down EPOR expression in both cell lines using two independent lentiviral shRNA sequences. EPOR expression was suppressed at both mRNA (Figure 1C) and protein levels (Figure 1D) by both shEPOR#1 and shEPOR#2, compared to the scrambled control (shSCR) at 72 hours in MDA-MB-231 cells and in MDA-MB-435 cells (data not shown). Addition of EPO resulted in lower activation of the PI3K/AKT pathway in EPOR-depleted MDA-MB-231 cells as shown by the lack of significant increase in pAKT in MDA-MB-231 cells (Figure 1E). Thus EPO can induce EPOR-dependent activation of the AKT signaling pathway in MDA-MB-231 cells. Addition of EPO to EPOR-depleted MDA-MB-231 cells did not result in a significant decrease in pERK compared to scrambled control cells (data not shown).

### EPOR knockdown abrogates human tumor cell growth

**Figure 2:**
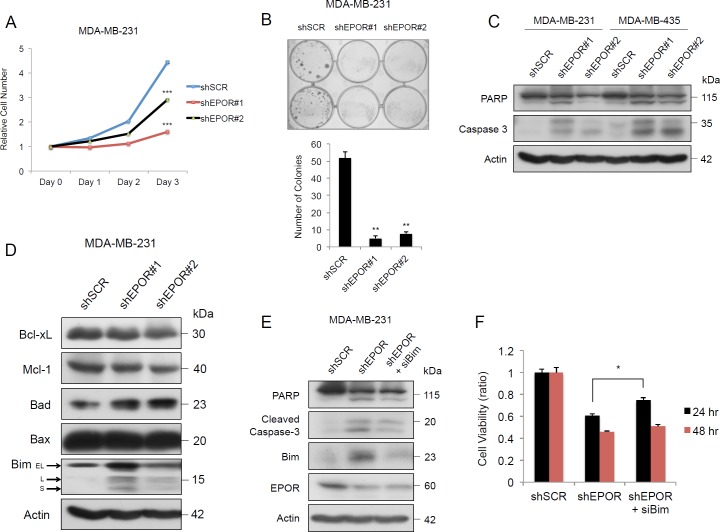
EPOR is essential for growth and prevents apoptosis in breast cancer cells **A**. Growth curve of MDA-MB-231 cells transduced with shSCR, shEPOR#1, or shEPOR#2 measured by MTT colorimetric assay to determine cell viability. shSCR *vs* shEPOR#1, ***p = 0.0003 at Day 3. shSCR *vs* shEPOR#2, ***p < 0.0001 at Day 3. **B**. Clonogenic assay of MDA-MB-231-shSCR, MDA-MB-231-shEPOR#1 and MDA-MB-231-shEPOR#2 cells. Colony number was quantified in three independent replicates after 5 days. shSCR *vs* shEPOR#1, **p = 0.002; shSCR *vs* shEPOR#2, **p = 0.0041. **C**. Immunoblots of PARP and Caspase 3 in MDA-MB-231-shSCR, MDA-MB-231-shEPOR#1 and MDA-MB-231-shEPOR#2 cells, and MDA-MB-435-shSCR, MDA-MB-435-shEPOR#1 and MDA-MB-435-shEPOR#2 cells harvested 72 hours after transduction. **D**. Immunoblots of apoptosis-related genes in MDA-MB-231-shSCR, MDA-MB-231-shEPOR#1 and MDA-MB-231-shEPOR#2 cells, harvested 72 hours after transduction. **E**. Immunoblots of cleaved forms of PARP and Caspase 3 in MDA-MB-231-shSCR, MDA-MB-231-shEPOR#1 and MDA-MB-231-shEPOR#1/siBim dual knockdown cells. Cell lysates were prepared 72 hours after knockdown. **F**. MTT activity of MDA-MB-231-shSCR, MDA-MB-231-shEPOR#1 and MDA-MB-231-shEPOR#1/siBim dual knockdown cells. Relative cell numbers were determined by MTT assay at both 24 and 48 hours. shEPOR#1 *vs* shEPOR#1/siBim at 24 hours, *p = 0.0121.

Using the MTT assay, EPOR-depleted MDA-MB-231 and MDA-MB-435 cells showed significantly reduced viable growth and colony formation compared to cells infected with scrambled shRNA (Figure 2A and 2B and Supplementary Figure S1A and S1B). These results indicate that the EPO-EPOR axis plays an important role in sustaining growth in these cell lines.

### EPOR knockdown induces apoptosis through Bim

Since EPO is a survival factor in erythroid progenitor cells, we investigated whether EPOR knockdown in cancer cell lines would lead to apoptosis. EPOR knockdown in both cell lines caused cleavage of PARP and caspase 3 (Figure 2C). EPOR knockdown increased the expression of BimL and BimS in both cell lines (Figure 2D and Supplementary Figure S1C). EPOR knockdown had no effect on Bax and Bcl-xL in either cell line; Mcl-1 expression was reduced in MDA-MB-231 cells but was unchanged in MDA-MB435 cells (Figure 2D and Supplementary Figure S1C). To verify that Bim is a mediator of apoptosis induced by EPOR depletion, we knocked down Bim by its specific siRNA and found that PARP and caspase 3 cleavage were decreased compared to cells with EPOR knockdown alone (Figure 2E). Relative cell viability in the dual EPOR/Bim knockdown was greater than in cells with EPOR knockdown alone after 24 hours (Figure 2F), suggesting that the survival of tumor cells was partially rescued by Bim depletion. The reversal of apoptosis caused by siBim was minimal after 48 hours, possibly due to degradation of siBim.

### EPO-induced MYC expression is mediated through PI3K/AKT and MAPK pathways

**Figure 3:**
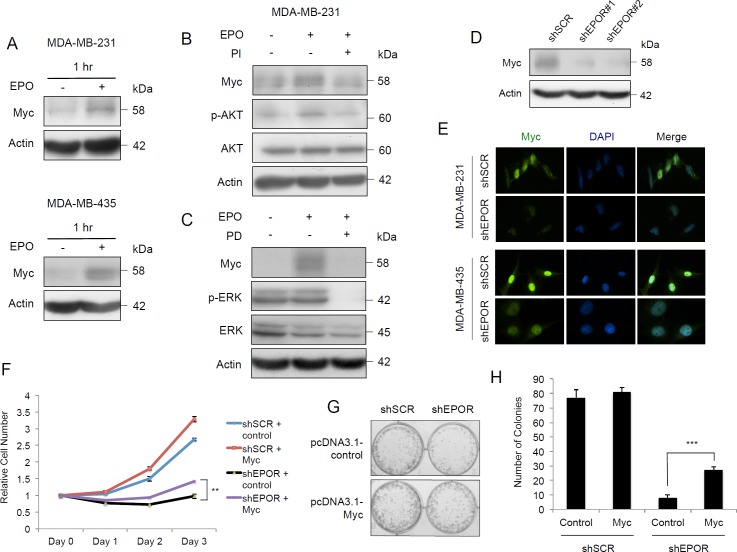
EPO induces Myc expression through PI3K/AKT and MAPK pathways and EPOR silencing decreases Myc in breast cancer cells **A**. Immunoblots of Myc after treatment with 10 U EPO/ml for 1 hour in MDA-MB-231 cells, and in MDA-MB-435 cells. **B**. Immunoblots of Myc, phospho-AKT, AKT, and actin in MDA-MB-231 cells after treatment with 10 U EPO/ml for 72 hours, and with addition of the PI3 kinase pathway inhibitor PI-103 (PI) or vehicle (DMSO) at 24 hours. **C**. Immunoblots of Myc, phospho-ERK1/2, ERK1/2, and actin in MDA-MB-231 cells after treatment with 10 U EPO/ml for 72 hours, and with addition of the Ras/MEK/ERK pathway inhibitor PD184352 (PD) or vehicle (DMSO) at 24 hours. **D**. Immunoblots of Myc in MDA-MB-231-shSCR, MDA-MB-231-shEPOR#1 and MDA-MB-231-shEPOR#2 cells, 72 hours following transduction. **E**. Immunofluorescence analysis of Myc in MDA-MB-231-shSCR, MDA-MB-231-shEPOR#2, MDA-MB-435-shSCR and MDA-MB-435-shEPOR#2 cells. Myc was detected using anti-Myc primary antibody and Alexa Fluor® 488 secondary antibody, and the nuclei were stained with DAPI (blue). Cells were fixed 72 hours after transduction. **F**. Representative growth curves of shSCR pcDNA3.1-control (shSCR-control), shSCR pcDNA3.1-Myc (shSCR-Myc), shEPOR pcDNA3.1-control (shEPOR-control) and shEPOR pcDNA3.1-Myc (shEPOR-Myc) MDA-MB-231 cells by MTT assay. shEPOR-control *vs* shEPOR-Myc, **p < 0.01. **G**. Clonogenic assay of shSCR-control, shSCR-Myc, shEPOR-control and shEPOR-Myc MDA-MB-231 cells assessed by colony count after 5 days. Images are representative of three independent experiments. **H**. Quantification of colonies in shSCR-control, shSCR-Myc, shEPOR-control and shEPOR-Myc MDA-MB-231 cells. Data are representative of three independent experiments. Viable colonies were identified as those with > 50 cells. shEPOR-control *vs* shEPOR-Myc, ***p = 0.0003.

We next sought to understand how the EPO/EPOR axis exerts these survival and growth effects, particularly in the context of breast tumorigenesis. EPO maintains c-Myc expression in murine erythroblasts throughout their differentiation into reticulocytes [14] and activates two distinct pathways required for the initiation and elongation of c-Myc in BaF3 immortalized murine pro-B-cells [15]. In human breast cancer, MYC is amplified and overexpressed in 12 to 100% of cases and is associated with an aggressive phenotype [16]. To investigate whether EPO can increase expression of Myc, the tumor cell lines were serum-starved for 24 hours, treated with 10 U EPO/ml for 1 hour and the levels of Myc were analyzed. Myc protein expression was increased after one hour of treatment with EPO in both cell lines (Figure 3A). As shown above, EPO activates the expression of PI3K/AKT and MAPK pathways in tumor cells (Figure 1A, and Figure 1B). To determine if EPO-dependent activation of *MYC* is mediated through these pathways, the PI3K inhibitor, PI-103 (PI), and the MEK inhibitor, PD184352 (PD) were tested in the presence or absence of 10 U EPO/ml. Inhibition of PI3K reversed the increase in pAKT and Myc protein expression produced by EPO treatment in MDA-MB-231 cells (Figure 3B) and in MDA-MB-435 cells (Supplementary Figure S2A). MEK inhibition reversed the increase in Myc expression produced by EPO addition (Figure 3C, Supplementary Figure S2B). Therefore, Myc induction caused by EPO is dependent on the activation of both the PI3K/AKT and MAPK pathways in both cell lines.

### EPOR knockdown causes downregulation of MYC expression

Next we investigated the effect of shEPOR on the endogenous level of Myc without pre-treatment by serum starvation or the addition of EPO. Transfection of the cells with shEPOR#1 and shEPOR#2 caused a decrease of Myc protein expression by 48 hours in both MDA-MB-231 (Figure 3D) and MDA-MB-435 cell lines (Supplementary Figure S2C). Thus EPO can induce Myc expression, whereas knockdown of EPOR reduces Myc expression independently of EPO.

Knockdown of EPOR reduced Myc protein expression in both the cytosolic and nuclear fractions in MDA-MB-231 cells (Supplementary Figure S2D), and in the nuclear fraction in MDA-MB-435 cells (Supplementary Figure S2E). This was confirmed by immunofluorescence which showed that Myc is located predominantly in the nucleus of both cell lines and that Myc expression is reduced in the nucleus of EPOR knockdown cells compared to the scrambled control (Figure 3E).

### Overexpression of MYC partially rescues loss of cell proliferation caused by EPOR downregulation

To further delineate the role of *MYC* regulation by EPOR, *MYC* was transiently transfected into MDA-MB-231 cells followed by lentiviral-mediated EPOR knockdown by its specific shRNA (Supplementary Figure S2F). After 3 days, the number of EPOR-depleted cells with overexpressed *MYC* (shEPOR-MYC) was significantly higher compared to EPOR knockdown alone (shEPOR-control) (Figure 3F). *MYC* overexpression enhanced colony formation and partially rescued them from the attenuated growth caused by *EPOR* knockdown (Figure 3G and 3H).

### EPOR knockdown reduces invasiveness in human tumor cell lines

**Figure 4:**
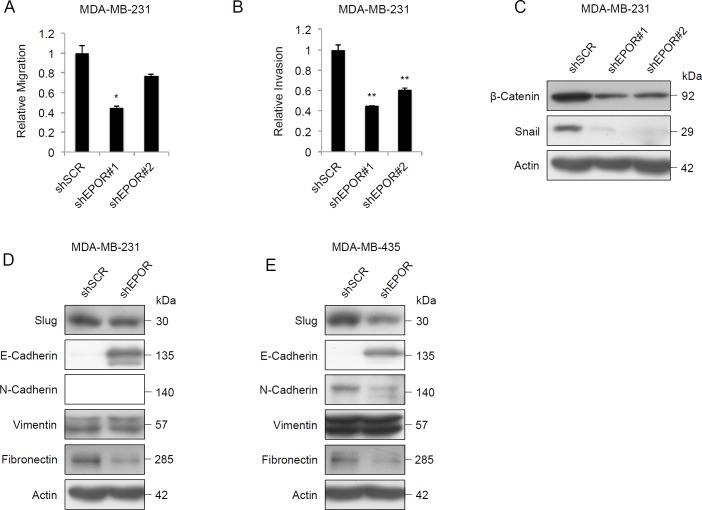
Effect of EPOR knockdown on migration, invasion and EMT protein expression **A**. *In vitro* migration in MDA-MB-231-shSCR, MDA-MB-231-shEPOR#1 and MDA-MB-231-shEPOR#2 breast tumor cells, measured as the relative migration through membrane inserts with 8 μm pore size, after 24 hours. Data shown are mean fold changes ± SEM for three independent replicates, *p < 0.05, paired t test. **B**. *In vitro* invasion in MDA-MB-231-shSCR, MDA-MB-231-shEPOR#1 and MDA-MB-231-shEPOR#2 cells, measured as the relative invasion through Matrigel-coated invasion chamber inserts with 8 μm pore size. Cells seeded in the upper chamber were allowed to migrate through a Matrigel-coated membrane for 24 hours using fetal bovine serum as chemoattractant. Data shown are mean fold changes ± SEM for three independent replicates, **p < 0.01, paired t test. **C**. Immunoblot of β-catenin and Snail in MDA-MB-231-shSCR, MDA-MB-231-shEPOR#1 and MDA-MB-231-shEPOR#2 cells. Total protein was harvested 72 hours after viral transduction. (D,E) Immunoblots of Slug, E-cadherin, N-cadherin, Vimentin and Fibronectin in **D**. MDA-MB-231-shSCR and MDA-MB-231-shEPOR#1 cells and in **E**. MDA-MB-435-shSCR and MDA-MB-435-shEPOR#1 cells. Total protein was harvested 72 hours after viral transduction.

We next investigated if EPOR regulates migration and invasion in tumor cell lines. EPOR knockdown caused a 20-60% decrease in cell migration (Figure 4A and Supplementary Figure S3A) and a 30-60% decrease in cell invasion (Figure 4B and Supplementary Figure S3B) in the cell lines. Knockdown of EPOR caused a decrease in β-catenin and Snail protein expression in both cell lines (Figure 4C and Supplementary Figure S3C), but there was no change in Twist expression (data not shown). Following fractionation into cytosolic and nuclear fractions, β-catenin reduction was more pronounced in the cytoplasmic fraction (Supplementary Figure 3D and 3E). We extended this analysis to a range of epithelial-mesenchymal transition (EMT) markers. After EPOR knockdown Slug and fibronectin expression was decreased in both cell lines, and E-cadherin protein expression increased in both cell lines (Figure 4D and 4E). N-cadherin expression decreased in MDA-MB-435 cells but was not detected in MDA-MB-231 cells. There was no change in vimentin expression. These results suggest that EPOR is required for cancer cell migration and invasion, and regulates the expression of proteins that are essential for EMT.

### Knockdown of EPOR reduces tumor growth *in vivo*

To investigate the role of EPOR in tumor development *in vivo*, MDA-MB-231-D3H2 cells harboring either a tet-on-inducible shEPOR (shEPOR) or a tet-on-inducible scrambled shSCR sequence (shSCR) were prepared and validated prior to subcutaneous (s.c.) injection in B6N nude female mice (Supplementary Figure S4A, S4B, S4C and S4D).

**Figure 5:**
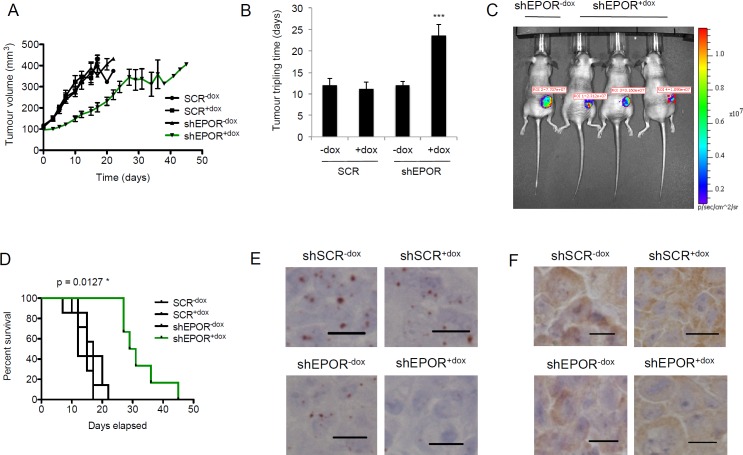
Effect of EPOR knockdown on breast tumor growth ***in vivo***. **A**. Tumor volume measurements of mice in the four subgroups, shSCR^−dox^ (*n* = 7), shSCR^+dox^ (*n* = 7), shEPOR^−dox^ (*n* = 7) and shEPOR^+dox^ (*n* = 6), following the commencement of doxycycline and EPO treatment at 100 mm^3^ tumor volume. **B**. Tumor tripling time in days for the tumor to grow from 100 mm^3^ to 300 mm^3^. ***p < 0.0001 for shEPOR^+dox^ *vs* shSCR^−dox^; shEPOR^+dox^ *vs* shSCR^+dox^; shEPOR^+dox^ *vs* shEPOR^−dox^; and shEPOR^+dox^ *vs* shEPOR^−dox^, Bonferroni's Multiple Comparison Test. All other comparisons between subgroups were non-significant (NS). **C**. *In Vivo* Imaging System (IVIS) time-matched images of one shEPOR^−dox^ mouse (left lane) and three shEPOR^+dox^ mice on Day 15. **D**. Kaplan-Meier plot of the survival time in shSCR^−dox^, shSCR^+dox^, shEPOR^−dox^ and shEPOR^+dox^ animals. Mice were culled when the tumor volume reached 400 mm^3^; *p = 0.0127, log-rank (Mantel-Cox) test. **E**. RNAscope^®^ analysis of *EPOR* expression in representative tumors from shSCR^−dox^, shSCR^+dox^, shEPOR^−dox^ and shEPOR^+dox^ mice. Scale bar represents 10 μm. **F**. Immunohistochemical analysis of EPOR expression, using the anti-EPOR antibody GM1201, in representative tumors from shSCR^−dox^, shSCR^+dox^, shEPOR^−dox^ and shEPOR^+dox^ mice. Images were taken at x40 magnification. Scale bar represents 10 μm.

To simulate treatment of anemia in cancer patients, all mice were treated with recombinant human EPO (500 U/kg) by intra-peritoneal injection twice per week. Tumor growth in the shEPOR^+dox^ subgroup was significantly reduced compared to the shSCR^−dox^, shSCR^+dox^ and shEPOR^−dox^ subgroups (Figure 5A). Doxycycline-induced knockdown of EPOR significantly increased tumor tripling time from 12.0±1.6 (shSCR^−dox^), 11.2±1.5 (shSCR^+dox^), 12.0±1.0 (shEPOR^−dox^) days to 23.6±2.6 (Figure 5B). IVIS imaging of time-matched shEPOR^−dox^ and shEPOR^+dox^ mice at Day 15 indicated a reduction in bioluminescence activity in the shEPOR^+dox^ mice (Figure 5C). Mice in the shEPOR^+dox^ subgroup had a significantly increased mean survival time to maximum tumor limit (30 days) compared to the other three subgroups (mean of 14.7 days) (Figure 5D).

Analysis of tumor material from each of the four subgroups by RNAscope^®^, using human *EPOR* probes, showed substantially lower expression of EPOR in the shEPOR^+dox^ subgroup compared to the other three subgroups (Figure 5E). Furthermore, immunohistochemical staining also confirmed reduced EPOR expression in tumors from shEPOR^+dox^ mice (Figure 5F).

The tumor-bearing mice showed small increases in red cell count, hemoglobin, and hematocrit, indicating that they had not become anemic over the experimental period (Supplementary Figure S4E). Imaging of turbo Red Fluorescence Protein (tRFP) showed consistent tRFP fluorescence in shEPOR^+dox^ mice whereas no tRFP signal was detected in shEPOR^−dox^ mice, (Supplementary Figure S4F), indicating that the dox-inducible shEPOR vector was functionally active *in vivo* for up to 7 weeks post transplantation.

## DISCUSSION

The JAK2/STAT5, PI3K/AKT and MEK/ERK signaling pathways are frequently altered in tumor cells and evidence that EPO induces these signal transduction cascades is accumulating [17–19]. Previously we showed that EPO-induced activation of the JAK2/STAT5, PI3K/AKT and Ras/ERK pathways promotes malignant cell behavior in a benign non-invasive rat cell line Rama 37 stably transfected with human EPOR [9]. Here we have investigated the effects of EPOR knockdown in two human breast cancer cell lines and in a xenotransplantation model designed to mimic EPO therapy in breast cancer patients. Given that EPO-induced signalling components are more readily detected in the Rama 37 cells overexpressing EPOR, the results of the two studies are in good agreement and corroborate the idea that the EPO-EPOR axis is important in breast cancer progression.

Knockdown of EPOR caused an increase in apoptotic activity in both MDA-MB-231 and MDA-MB-435 cell lines, as demonstrated by increased cleavage of PARP and caspase 3. Moreover EPOR knockdown caused an increase in the expression of BimL and BimS, which are BH3-only proteins of the Bcl-2 family that regulate the cell death program *via* the mitochondrial apoptotic pathway. Bim binds to pro-survival proteins such as Mcl-1 and releases Bax to initiate cell death [20, 21]. This may explain our observation that the level of Bax protein expression was not changed upon silencing of EPOR, in spite of the increase in apoptosis. Bim is also regulated in part by post-translational modification [22]. In particular ERK1/2 phosphorylates BimEL causing rapid degradation *via* the proteasome pathway, whereas ERK1/2 does not contribute to the degradation of BimS or BimL [22]. EPOR silencing causes impairment of the MAPK pathway leading to increased stability of Bim. In cells with combined EPOR and Bim downregulation, apoptosis was decreased compared to cells with EPOR downregulation alone. This confirms that Bim is involved in apoptosis and that EPOR-mediated signaling contributes to survival in both cell lines. However the apoptotic effect after EPOR knockdown was not completely reversed suggesting that other mediators of apoptosis are involved.

The PI3K pathway mediates many cellular processes including proliferation, differentiation, ribosomal biosynthesis and mitochondrial function, and is frequently dysregulated in cancer. c-Myc is highly expressed in many human cancers and is associated with poor clinical outcome [23]. EPO increases *MYC* expression in erythroid progenitor cells [14] and we found that EPO increases *MYC* expression in both breast cancer cell lines. Moreover AKT, a canonical downstream effector of PI3K, and MAPK can affect transcription and degradation of Myc [24] so we questioned whether activation of the PI3K/AKT and MAPK pathways and the increase in Myc are causally linked. PI103, a potent inhibitor of PI3K, and PD184352, a specific inhibitor of MEK, were each able to block the increase in Myc expression, indicating that both pathways are involved in the EPO-induced increase in Myc found in both cell lines.

EPO has been reported to increase lymph node lymphangiogenesis and lymph node tumor metastasis in a mouse model of breast cancer [25]. These effects were associated with increased migration, capillary-like tube formation, and dose- and time-dependent proliferation of human lymphatic endothelial cells. Interestingly these effects were abrogated by co-treatment with specific inhibitors of PI3K or MAPK, under conditions in which EPO increased AKT and ERK1/2 phosphorylation and are consistent with our current data.

Lentiviral-mediated silencing of EPOR inhibited activation of the PI3K/AKT and MAPK pathways, leading to downregulation of *MYC* and reduced cell proliferation. Overexpression of *MYC* partially rescued the decrease in proliferation caused by *EPOR* silencing, indicating an overlap in the pathways downstream of *EPOR* signaling and Myc activation, and that Myc is important in EPOR-mediated cell survival. If this observation *in vitro* holds *in vivo* it would imply that EPO-induced signaling interacts with Myc, which is frequently amplified and over-expressed in breast tumors [16].

The epithelial-mesenchymal transition (EMT) is widely held to be critical for tumor invasiveness. In this process epithelial cells undergo multiple biochemical changes to develop a mesenchymal phenotype, which generally exhibits enhanced migratory capacity, invasiveness, elevated resistance to apoptosis, and increased production of extracellular matrix components [26] Here, we showed that EPOR knockdown caused both tumor cell lines to become more epithelial-like. The endogenous levels of E-cadherin, a transmembrane protein that controls homophilic cell-cell adhesion and maintains cells in an epithelial phenotype [27] were low, but increased after EPOR knockdown, accompanied by a decrease in N-cadherin. No N-cadherin expression could be detected in the MDA-MB-231 cells, consistent with previous data [28].

E-cadherin and N-cadherin appear to have a reciprocal relationship. N-cadherin is expressed primarily in tissues of mesenchymal origin, and upregulation of N-cadherin enhances cell invasiveness and mobility [29]. Switching from E-cadherin to N-cadherin promotes tumor progression and correlates with multiple clinical outcomes [30] [31]. The increase in E-cadherin may also be caused by the decrease in Snail, a transcriptional repressor of E-cadherin. After EPOR knockdown β-catenin was decreased in the nuclear fraction, reducing its binding to transcription factors of the T-cell factor (TCF) family and its capacity to modulate the downstream target genes involved in EMT [30].

**Figure 6:**
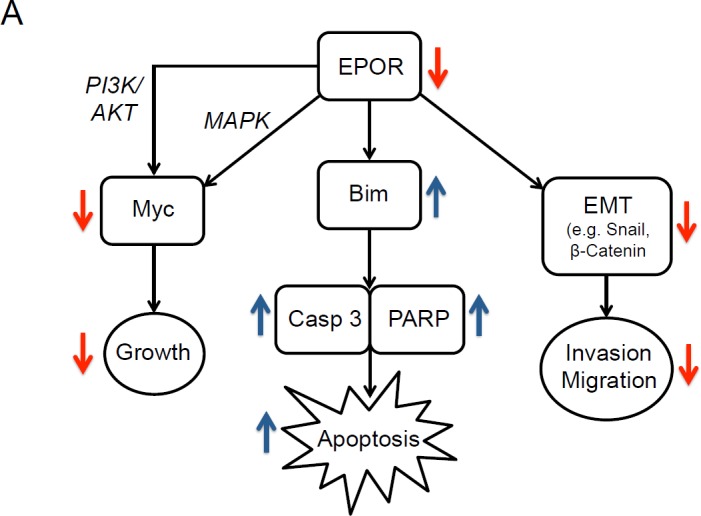
Effects of knockdown of EPOR in breast cancer cell lines Schematic diagram illustrating effects of EPOR depletion on downstream signaling proteins and on breast cancer cell growth, apoptosis, invasion and migration.

The effects of EPOR knockdown on both cell lines are summarized in Figure 6. After EPOR knockdown Myc is decreased, and is associated with decreased cell growth, increased apoptosis, and changes in EMT-related proteins linked to decreases in invasive and migratory capacity.

To investigate the role of EPOR in breast tumor progression we used a murine xenotransplantation model which simulated EPO therapy in cancer patients. Tumor growth in the MDA-MB-231-D3H2-shEPOR subgroup treated with doxycycline (shSCR^+dox^) was dramatically reduced and showed substantially lower expression of *EPOR* by *in situ* hybridization than the control subgroups, indicating that EPO-EPOR axis is active in breast tumor progression in this model.

Cell type-specific differences in EPOR signalling between primary erythroid and H838 lung cancer cells have recently been defined using a systems biology approach [32]. Seven cell type-specific parameters including nuclear location, translocation of STAT5 and target gene induction were identified. The model also predicted that JAK2 inhibition in combination with EPO treatment would selectively inhibit H838 cells compared to erythroid cells. This prediction is of particular interest because the JAK2 inhibitor Fedratinib was found to be synergistic with chemotherapy for breast tumor-initiating cells in autochthonous genetically engineered murine models [24]. Furthermore the efficacy of the JAK2 inhibitor Ruxolitinib is currently being investigated in combination with cisplatin in clinical trials in non-small cell lung carcinoma (NSCLC) [33].

## MATERIALS AND METHODS

### Cell lines

Human cancer cell lines MDA-MB-231 and MDA-MB-435 and the viral packaging cell line 293T were obtained from American Type Culture Collection (Manassas, VA). Both cancer cell lines are characterised by the lack of expression of ERα, PR and HER2 i.e. they are classified as triple negative. The origin of MDA-MB-435 is controversial, having been classified both as a breast cancer and as a melanoma cell line [34].

### Cell culture

Cell lines were maintained in Dulbecco's modified Eagle medium (DMEM) with high glucose and L-glutamine (PAA Laboratories) supplemented with 10% fetal bovine serum, 100 U/ml penicillin, and 100 μg/ml streptomycin. The cells were routinely cultured in a humidified incubator at 37°C supplemented with 5% CO_2_. Before stimulation with EPO, cells were serum-starved for 24 hours.

### Recombinant DNA constructs

#### Plasmids

pLKO.1-shEPOR#1 and pLKO.1-shEPOR#2 (clone IDs NM_000121.2-539s1c1 and NM_000121.2-1399s1c1 respectively) were purchased from Sigma. pLKO.1-scramble (SCR, #1864) and pcDNA3-cMyc (#16011) were from Addgene, and siBim (clone ID SI02655359) was from Qiagen. Scramble siRNA sequences: sense 5′-UUGUACGGCAUCAGCGUUAdTdT-3′; and antisense 5′-UUACGCUGAUGCCGUACAAdTdT-3′ were from Amaxa GmbH.

### Transfection and viral infection

Genejuice^®^ (Novagen) was used for transfection of expression plasmids or shRNA. For generation of cell lines with EPOR knockdown, 293T cells were first transfected with shRNA. Viral particle-containing medium was harvested 48 hours after transfection and used to transduce target breast cancer cells supplemented with 6 μg/ml polybrene. Four hours after infection, cells were refreshed with complete medium and a further cycle of infection was performed 24 hours later. Equivalent amounts of viral particles from the same batch of medium were used to achieve similar knockdowns to enable comparison of different treatments within a given experiment. For siRNA transfection, siPORT NeoFX transfection agent (Ambion, Life Technologies) was used according to the manufacturer's instructions.

### Quantitative RT-PCR

Total RNA was extracted using TRIzol (Invitrogen). The first-strand cDNA was synthesized from 1 μg RNA using the GeneAmp RNA PCR kit (Applied Biosystems). TaqMan probes were used for quantitative RT-PCR: *EPOR* (Hs00181092_1), 18S (4319413E) and analyzed using the ABI 7500H Light Cycler (Applied Biosystems).

### Inhibitors

PD184352 (a gift from Dr James Murray) and PI103 (Merck) were reconstituted in DMSO.

### Western blot

Cells were lysed in RIPA buffer [50 mM Tris-HCl (pH 8.0), 150 mM NaCl, 1% (v/v) NP-40, 0.5% (w/v) deoxycholate, and 0.1% (w/v) SDS, supplemented with phenylmethylsulfonyl fluoride, sodium orthovanadate, and a cocktail of protease inhibitors. The N-Per kit (Thermo Fisher Scientific) was used for cellular fractionation. Protein concentrations of the cell lysates were measured by the DC^TM^ Protein assay (Bio-Rad Laboratories).

### Sources of antibodies used in western blots

Mouse monoclonal antibodies against tubulin and actin were from Sigma and anti-phospho-ERK from Cell Signaling Technology. Polyclonal antibodies for the EMT markers, Bcl-2 family members, PARP, cleaved caspase 3, ERK, phospho-ERK, AKT, phospho-AKT (Serine 473), were from Cell Signaling Technology. Anti-TATA binding protein (TBP) antibody was from Abcam. Antibodies for Myc and EPOR (C20) were from Santa Cruz Biotechnology. For immunohistochemistry, the EPOR antibody GM1201 was used (Aldevron GmbH).

### Fluorescence microscopy

Cells grown on coverslips were fixed with ice-cold methanol and staining performed as described previously [35]. Myc was visualised using an anti-Myc primary antibody and Alexa Fluor^®^ 488 secondary antibody, and the nucleus counterstained with DAPI. The cell staining was visualized using an Eclipse Ti-S microscope (Nikon).

### Quantitative RT-PCR

Total RNA was extracted using TRIzol (Invitrogen). The first-strand cDNA was synthesized from 1 μg RNA using the GeneAmp RNA PCR kit (Applied Biosystems). TaqMan probes were used for quantitative RT-PCR: *EPOR* (Hs00181092_1), 18S (4319413E) and analyzed using the ABI 7500H Light Cycler (Applied Biosystems).

### Invasion and migration assays

Invasion was measured using Matrigel-coated multi-well inserts as previously described [9]. Matrigel-coated invasion chambers (6.4-mm diameter: 8-μm pore size; BD Biosciences) were used to assess the invasive capacity of MDA-MB-231-shSCR, MDA-MB-231-shEPOR#1 and MDA-MB-231-shEPOR#2 breast tumor cells. Briefly, 1 × 10^5^ cells were resuspended in serum-free, phenol-red free media and placed in the upper chambers. The cultures were incubated for 18 hours at 37°C in a 5% (v/v) CO_2_ atmosphere. The upper surfaces of the filters were wiped clean of cells and the filters were fixed by immersion in 100% (v/v) methanol and stained in 0.5% crystal violet for 25 minutes. Each membrane was washed in running distilled water and left to air-dry. After washing with sodium citrate/ethanol for 30 minutes, 200 μL of the solution were transferred into a 96-well plate and read at 570 nm in a Tecan plate reader. The percentage of invasion of each cell type was normalized using the percentage of invasion of MDA-MB-231-shSCR as 100%.

The migration assay was similar to the invasion assay except that the insert membrane was not coated with Matrigel. The percentage of migration of each cell type was normalized using the percentage of invasion of MDA-MB-231-shSCR as 100%.

### Viability and clonogenic assays

To assess cell viability, MTT assays were performed. Briefly, 4 × 10^3^ cells per well were seeded in a 96-well plate after infection, and allowed to grow until the time points shown prior to the replacement with 100 μL fresh medium containing 10 μL of 5 mg/mL MTT. After incubation for 3 hours the crystallized MTT precipitates in each well were dissolved in DMSO and the absorbance was measured at 570 nm.

Cell clonogenicity was measured by seeding cells in 6-well plates.

### Xenotransplantation model

The role of EPOR in tumor development was investigated in a xenotransplantation model using immunocompromised female B6.Cg/N-Foxn1^nu/nu^ nude mice. Mice were s.c. injected with either control MDA-MB-231-D3H2-shSCR (shSCR) or MDA-MB-231-D3H2-shEPOR (shEPOR) cells. When the tumor volume reached 100 mm^3^ all mice were injected intraperitoneally twice weekly with EPO and EPOR-knockdown was initiated by *ad libitum* consumption of drinking water containing 1 μg/ml doxycycline and 50 mg/ml sucrose.

### Immunohistochemistry

Tumor samples were fixed in formal saline (Premier Scientific) for 24 hours and processed to paraffin wax. Serial sections were cut at 4 μm, air-dried overnight and dewaxed. Antigen retrieval was performed by pressure-cooking for 2 minutes in TE buffer pH 9.0 and samples stained using the GM1201 anti-EPOR antibody at 4 μg/ml overnight at 4°C. Localisation was performed using rabbit anti-rat IgG (Thermo Fisher Scientific) at 10 μg/ml for 30 minutes at ambient temperature and Rabbit Envision/Diaminobenzidine (Dako). Sections were counterstained in Meyer's hematoxylin.

### *In Situ* hybridization

Serial sections from tumors were stained for EPOR mRNA using chromogenic RNAscope^®^ (Advanced Cell Diagnostics). After being air-dried overnight, sections were baked at 60°C for 1 hour, de-waxed and air-dried before pre-treatments. For all probes the sections were subjected to a mild pre-treatment protocol and RNAscope^®^ probes Hs-EPOR and a positive control probe. Detection of specific probe binding sites was performed with RNAscope^®^ 2.0 HD Reagent kit. All sections were counterstained in Meyer's hematoxylin.

All experiments were performed in at least three independent experiments. The statistical tests used in each graph are described in the Figure legends. In all experiments, statistical significance was indicated as: *p* < 0.05 *; *p* < 0.01 **; *p* < 0.001 ***.

European Commission FP7 (EpoCan) 282551 (TRL, KBM).

Invest NI RD0914223 (TRL, KBM).

## SUPPLEMENTARY MATERIALS FIGURES AND TABLES



## References

[1] Caro JJ, Salas M, Ward A, Goss G (2001). Anemia as an independent prognostic factor for survival in patients with cancer: a systemic, quantitative review. Cancer.

[2] Henke M, Laszig R, Rube C, Schafer U, Haase KD, Schilcher B, Mose S, Beer KT, Burger U, Dougherty C, Frommhold H (2003). Erythropoietin to treat head and neck cancer patients with anaemia undergoing radiotherapy: randomised, double-blind, placebo-controlled trial. Lancet.

[3] Leyland-Jones B, Investigators B, Study G (2003). Breast cancer trial with erythropoietin terminated unexpectedly. Lancet Oncol.

[4] Sytkowski AJ (2007). Does erythropoietin have a dark side? Epo signaling and cancer cells. Sci STKE.

[5] Brines M, Cerami A (2005). Emerging biological roles for erythropoietin in the nervous system. Nat Rev Neurosci.

[6] Ghezzi P, Bernaudin M, Bianchi R, Blomgren K, Brines M, Campana W, Cavaletti G, Cerami A, Chopp M, Coleman T, Digicaylioglu M, Ehrenreich H, Erbayraktar S (2010). Erythropoietin: not just about erythropoiesis. Lancet.

[7] Westenfelder C, Baranowski RL (2000). Erythropoietin stimulates proliferation of human renal carcinoma cells. Kidney Int.

[8] Lopez TV, Lappin TR, Maxwell P, Shi Z, Lopez-Marure R, Aguilar C, Rocha-Zavaleta L (2011). Autocrine/paracrine erythropoietin signalling promotes JAK/STAT-dependent proliferation of human cervical cancer cells. Int J Cancer.

[9] Shi Z, Hodges VM, Dunlop EA, Percy MJ, Maxwell AP, El-Tanani M, Lappin TR (2010). Erythropoietin-induced activation of the JAK2/STAT5, PI3K/Akt, and Ras/ERK pathways promotes malignant cell behavior in a modified breast cancer cell line. Mol Cancer Res.

[10] Um M, Gross AW, Lodish HF (2007). A “classical” homodimeric erythropoietin receptor is essential for the antiapoptotic effects of erythropoietin on differentiated neuroblastoma SH-SY5Y and pheochromocytoma PC-12 cells. Cell Signal.

[11] Noguchi CT, Asavaritikrai P, Teng R, Jia Y (2007). Role of erythropoietin in the brain. Crit Rev Oncol Hematol.

[12] Larsson AM, Jirstrom K, Fredlund E, Nilsson S, Ryden L, Landberg G, Pahlman S (2009). Erythropoietin receptor expression and correlation to tamoxifen response and prognosis in breast cancer. Clin Cancer Res.

[13] Liang K, Esteva FJ, Albarracin C, Stemke-Hale K, Lu Y, Bianchini G, Yang CY, Li Y, Li X, Chen CT, Mills GB, Hortobagyi GN, Mendelsohn J (2010). Recombinant human erythropoietin antagonizes trastuzumab treatment of breast cancer cells via Jak2-mediated Src activation and PTEN inactivation. Cancer Cell.

[14] Bondurant MC, Yamashita T, Muta K, Krantz SB, Koury MJ (1996). C-myc expression affects proliferation but not terminal differentiation or survival of explanted erythroid progenitor cells. J Cell Physiol.

[15] Chen C, Sytkowski AJ (2001). Erythropoietin activates two distinct signaling pathways required for the initiation and the elongation of c-myc. J Biol Chem.

[16] Dueck AC, Reinholz MM, Geiger XJ, Tenner K, Ballman K, Jenkins RB, Riehle D, Chen B, McCullough AE, Davidson NE, Martino S, Sledge GW, Kaufman PA (2013). Impact of c-MYC protein expression on outcome of patients with early-stage HER2+ breast cancer treated with adjuvant trastuzumab NCCTG (alliance) N9831. Clin Cancer Res.

[17] Liu P, Cheng H, Roberts TM, Zhao JJ (2009). Targeting the phosphoinositide 3-kinase pathway in cancer. Nat Rev Drug Discov.

[18] Schild C, Wirth M, Reichert M, Schmid RM, Saur D, Schneider G (2009). PI3K signaling maintains c-myc expression to regulate transcription of E2F1 in pancreatic cancer cells. Mol Carcinog.

[19] Tsai WB, Aiba I, Long Y, Lin HK, Feun L, Savaraj N, Kuo MT (2012). Activation of Ras/PI3K/ERK pathway induces c-Myc stabilization to upregulate argininosuccinate synthetase, leading to arginine deiminase resistance in melanoma cells. Cancer Res.

[20] Marani M, Tenev T, Hancock D, Downward J, Lemoine NR (2002). Identification of novel isoforms of the BH3 domain protein Bim which directly activate Bax to trigger apoptosis. Mol Cell Biol.

[21] Gomez-Bougie P, Bataille R, Amiot M (2005). Endogenous association of Bim BH3-only protein with Mcl-1, Bcl-xL and Bcl-2 on mitochondria in human B cells. Eur J Immunol.

[22] Ley R, Ewings KE, Hadfield K, Cook SJ (2005). Regulatory phosphorylation of Bim: sorting out the ERK from the JNK. Cell Death Differ.

[23] Efstratiadis A, Szabolcs M (2007). Notch, Myc and breast cancer. Cell Cycle.

[24] Zhou B, Damrauer JS, Bailey ST, Hadzic T, Jeong Y, Clark K, Fan C, Murphy L, Lee CY, Troester MA, Miller CR, Jin J, Darr D (2014). Erythropoietin promotes breast tumorigenesis through tumor-initiating cell self-renewal. J Clin Invest.

[25] Lee AS, Kim DH, Lee JE, Jung YJ, Kang KP, Lee S, Park SK, Kwak JY, Lee SY, Lim ST, Sung MJ, Yoon SR, Kim W (2011). Erythropoietin induces lymph node lymphangiogenesis and lymph node tumor metastasis. Cancer Res.

[26] Kalluri R, Neilson EG (2003). Epithelial-mesenchymal transition and its implications for fibrosis. J Clin Invest.

[27] Baranwal S, Alahari SK (2009). Molecular mechanisms controlling E-cadherin expression in breast cancer. Biochem Biophys Res Commun.

[28] Nieman MT, Prudoff RS, Johnson KR, Wheelock MJ (1999). N-cadherin promotes motility in human breast cancer cells regardless of their E-cadherin expression. J Cell Biol.

[29] Chung S, Yao J, Suyama K, Bajaj S, Qian X, Loudig OD, Eugenin EA, Phillips GR, Hazan RB (2013). N-cadherin regulates mammary tumor cell migration through Akt3 suppression. Oncogene.

[30] Cowin P, Rowlands TM, Hatsell SJ (2005). Cadherins and catenins in breast cancer. Curr Opin Cell Biol.

[31] Kotb AM, Hierholzer A, Kemler R (2011). Replacement of E-cadherin by N-cadherin in the mammary gland leads to fibrocystic changes and tumor formation. Breast Cancer Res.

[32] Merkle R, Steiert B, Salopiata F, Depner S, Raue A, Iwamoto N, Schelker M, Hass H, Wasch M, Bohm ME, Mucke O, Lipka DB, Plass C (2016). Identification of Cell Type-Specific Differences in Erythropoietin Receptor Signaling in Primary Erythroid and Lung Cancer Cells. PLoS Comput Biol.

[33] Buchert M, Burns CJ, Ernst M (2016). Targeting JAK kinase in solid tumors: emerging opportunities and challenges. Oncogene.

[34] Holliday DL, Speirs V (2011). Choosing the right cell line for breast cancer research. Breast Cancer Res.

[35] Chan KK, Shen L, Au WY, Yuen HF, Wong KY, Guo T, Wong ML, Shimizu N, Tsuchiyama J, Kwong YL, Liang RH, Srivastava G (2010). Interleukin-2 induces NF-kappaB activation through BCL10 and affects its subcellular localization in natural killer lymphoma cells. J Pathol.

