# Supplementary material for: Erythropoietin drives breast cancer progression by activation of its receptor EPOR
Source: Oncotarget. 2017 Mar 18;8(24):38251–63. doi: 10.18632/oncotarget.16368 (PMC5503530; doi:10.18632/oncotarget.16368)
Supplement: Supplementary file 1 [file oncotarget-08-38251-s001.pdf]

# Erythropoietin drives breast cancer progression by activation of its receptor EPOR

## Supplementary Material

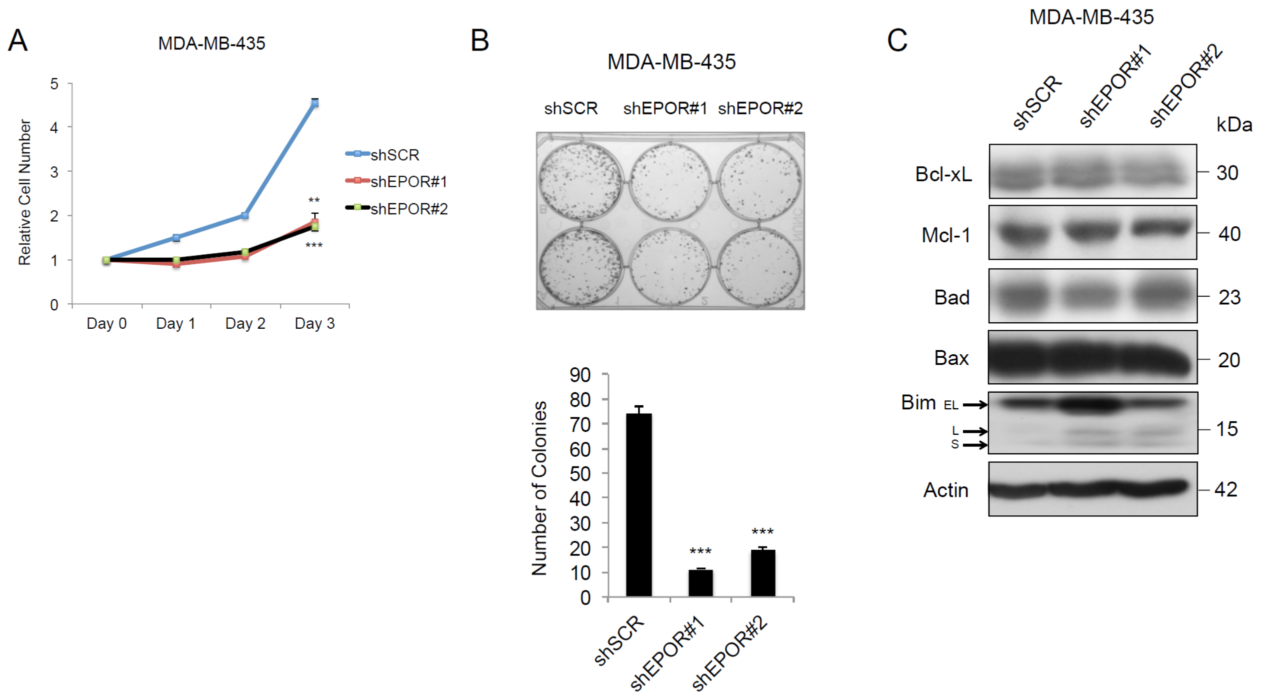


**Supplementary Figure 1: EPOR is essential for growth and modulates apoptosis-related proteins in breast cancer cells.** (A) Growth curve of MDA-MB-435 cells transduced with shSCR (MDA-MB-435-shSCR), shEPOR#1 (MDA-MB-435-shEPOR#1) or shEPOR#2 (MDA-MB-435-shEPOR#2) measured by MTT colorimetric assay to determine cell viability. shSCR vs shEPOR#1, **p = 0.005 at Day 3. shSCR vs shEPOR#2, ***p = 0.0005 at Day 3, paired t test data shown are means ± SEM. (B) Clonogenic assay of MDA-MB-435-shSCR, MDA-MB-435-shEPOR#1 and MDA-MB-435-shEPOR#2 cells. Colony number was quantified in four independent replicates after 5 days. shSCR vs shEPOR#1, ***p = 0.0002; shSCR vs shEPOR#2, ***p = 0.0002, paired t test. (C) Immunoblots of apoptosis-related genes, Bcl-xL, Mcl-1, Bad, Bax and the Bim isoforms $Bim_{EL}$, $Bim_L$ and $Bim_S$ in MDA-MB-435-shSCR, MDA-MB-435-shEPOR#1 and MDA-MB-435-shEPOR#2 cells, harvested 72 hours after transduction. Actin was used as loading control.

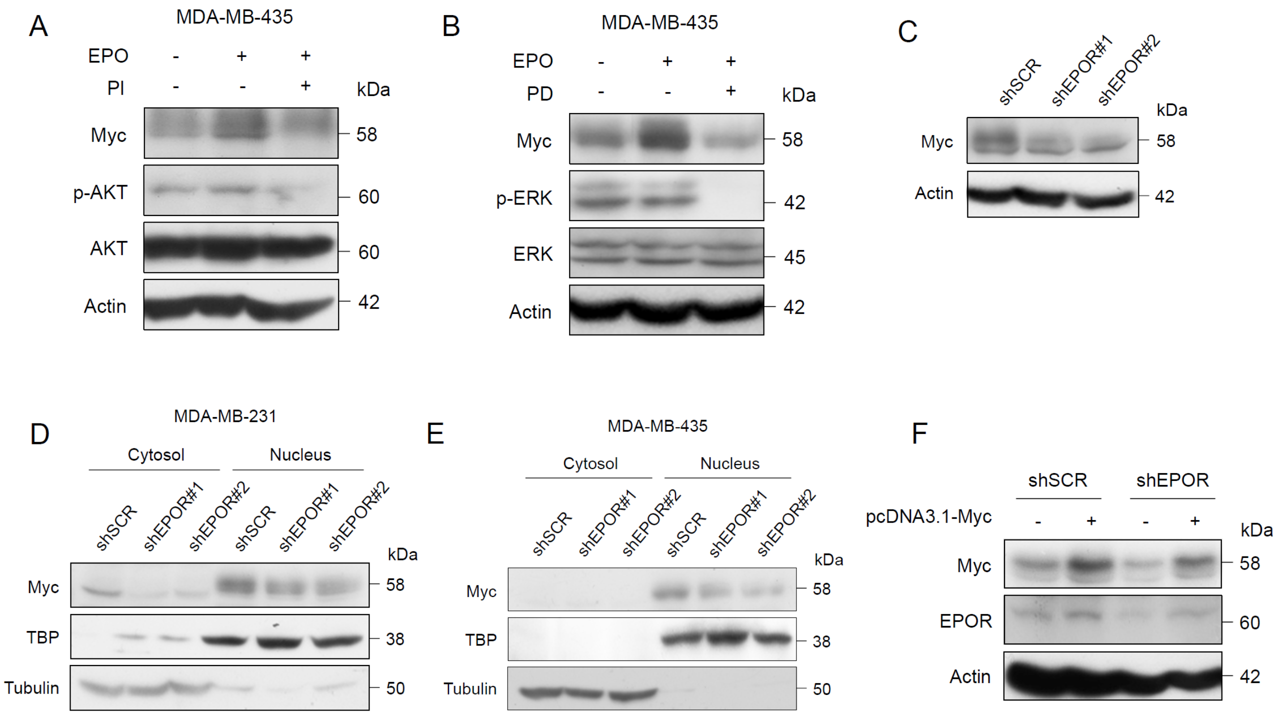


**Supplementary Figure 2: EPO induces Myc expression through PI3K/AKT and MAPK pathways and EPOR silencing decreases Myc expression in breast cancer cells.** (A) Immunoblots of Myc, phospho-AKT, AKT, and actin in MDA-MB-435 cells after treatment with 10 U EPO/ml for 72 hours, and with the addition of the PI3 kinase pathway inhibitor PI-103 (PI) or vehicle (DMSO) at 24 hours. (B) Immunoblots of Myc, phospho-ERK1/2, ERK1/2, and actin in MDA-MB-435 cells after treatment with 10 U EPO/ml for 72 hours, and with the addition of the Ras/MEK/ERK pathway inhibitor PD184352 (PD) or vehicle (DMSO) at 24 hours. (C) Western blot analysis of Myc in MDA-MB-435-shSCR, MDA-MB-435-shEPOR#1 and MDA-MB-435-shEPOR#2 cells, 72 hours following transduction. (D) Immunoblots of Myc in cytosolic and nuclear fractions from MDA-MB-231-shSCR, MDA-MB-231-shEPOR#1 and MDA-MB-231-shEPOR#2 cells. Protein was harvested 72 hours after transduction. TATA box-binding protein (TBP) was used to normalize the nuclear fractions and tubulin for the cytosolic fractions. (E) Immunoblots of Myc in cytosolic and nuclear fractions from MDA-MB-435-shSCR, MDA-MB-435-shEPOR#1 and MDA-MB-435-shEPOR#2 cells. Protein was harvested 72 hours after transduction. TATA box-binding protein (TBP) was used to normalize the nuclear fractions and tubulin for the cytosolic fractions. (F) Immunoblots of Myc and EPOR in MDA-MB-231-shSCR (shSCR) and MDA-MB-231-shEPOR (shEPOR) cells, either expressing vector control (-) or Myc (+), generated by transfection of pcDNA3.1-control and pcDNA3.1-Myc, and harvested after 72 hours.

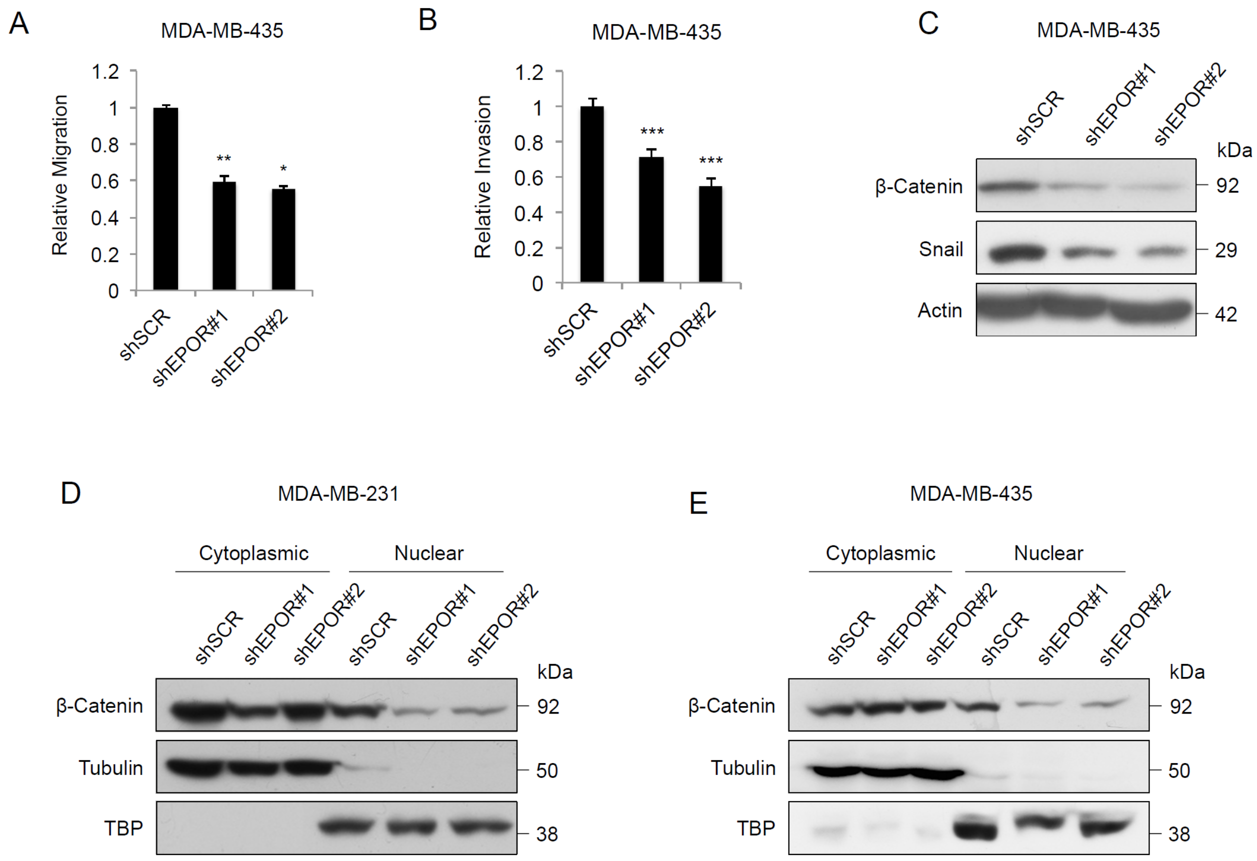


**Supplementary Figure 3: Effect of EPOR knockdown on migration, invasion and EMT protein expression.** (A) *In vitro* migration in MDA-MB-435-shSCR, MDA-MB-435-shEPOR#1 and MDA-MB-435-shEPOR#2 breast tumor cells, measured as the relative migration through membrane inserts with 8 µm pore size, after 24 hours. The ratio of migration of MDA-MB-435-shEPOR#1 and MDA-MB-435-shEPOR#2 cells was normalized to the average migration in MDA-MB-435-shSCR cells, which was set to 1. MDA-MB-435-shSCR vs MDA-MB-435-shEPOR#1, $^{**}p < 0.01$; MDA-MB-435-shSCR vs MDA-MB-435-shEPOR#2, $^{*}p < 0.05$, paired t test. Data shown are means ± SEM. (B) *In vitro* invasion in MDA-MB-435-shSCR, MDA-MB-435-shEPOR#1 and MDA-MB-435-shEPOR#2 breast tumor cells, measured as the relative invasion through Matrigel-coated invasion chamber inserts with 8 µm pore size. Cells seeded in the upper chamber were allowed to migrate through a Matrigel-coated membrane for 24 hours using fetal bovine serum as chemoattractant. The ratio of invasion of MDA-MB-435-shEPOR#1 and MDA-MB-435-shEPOR#2 cells was normalized to the average invasion in MDA-MB-435-shSCR cells, which was set to 1. MDA-MB-435-shSCR vs MDA-MB-435-shEPOR#1, $^{***}p < 0.001$; MDA-MB-435-shSCR vs MDA-MB-435-shEPOR#2, $^{***}p < 0.001$, paired t test. (C) Immunoblots of β-catenin and Snail in MDA-MB-435-shSCR, MDA-MB-435-shEPOR#1 and MDA-MB-435-shEPOR#2 breast cells. Total protein was harvested 72 hours after viral transduction. Actin was used as loading control. (D) Immunoblots of β-catenin in cytosolic and nuclear fractions from MDA-MB-231-shSCR, MDA-MB-231-shEPOR#1 and MDA-MB-231-shEPOR#2 cells. Protein was harvested 72 hours after transduction. TATA box-binding protein (TBP) was used to normalize the nuclear fractions and tubulin for the cytosolic fractions. (E) Western blot analysis of β-catenin in cytoplasmic and nuclear fractions from MDA-MB-435-shSCR, MDA-MB-435-shEPOR#1 and MDA-MB-435-shEPOR#2 cells. Protein was harvested 72 hours after transduction. TATA box-binding protein (TBP) was used to normalize the nuclear fractions and tubulin for the cytosolic fractions.

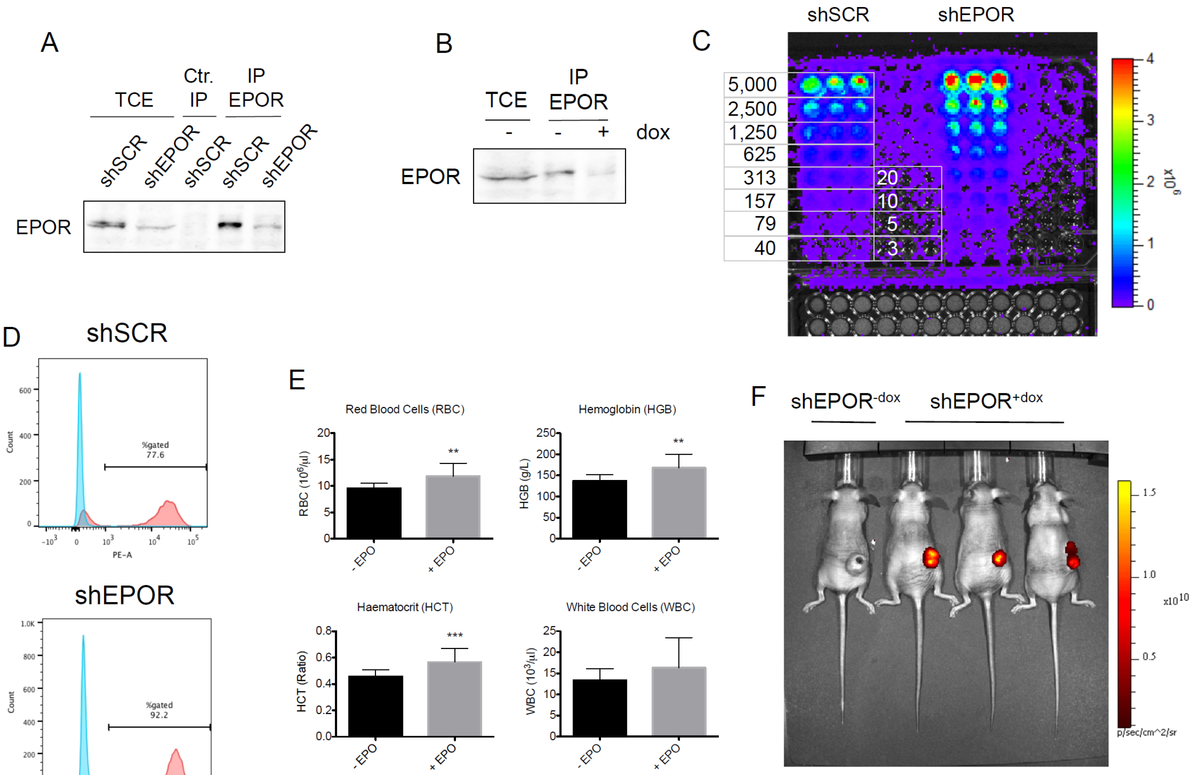


**Supplementary Figure 4: Effect of EPOR knockdown on breast tumor growth in vivo.** (A) Immunoprecipitation (IP) analysis of MDA-MB-231-D3H2-shSCR (shSCR) and MDA-MB-231-D3H2-shEPOR (shEPOR) protein extracts following 72 hours treatment with 1 μg/ml doxycycline. EPOR was immunoprecipitated with a 1:1 mix of GM1202 and GM1203 antibodies covalently coupled to Protein A/G Agarose (Pierce, Thermo Scientific) as described [55]. GM1201 was used for Western blot detection and anti-HA antibody 12CA5 for the negative control IP (Ctr. IP). TCE (total cell extract): 10% of the protein input for the IP was loaded. (B) Immunoprecipitation (IP) analysis of MDA-MB-231-D3H2-shEPOR (shEPOR) protein extracts following either no treatment (-) or treatment with 1 μg/ml doxycycline for 72 hours. EPOR was immunoprecipitated with a 1:1 mix of GM1202 and GM1203 antibodies covalently coupled to Protein A/G Agarose. GM1201 was used for Western blot detection. TCE (total cell extract): 10% of the protein input for the IP was loaded. (C) IVIS analysis of MDA-MB-231-D3H2-shSCR and MDA-MB-231-D3H2-shEPOR cells at serial dilutions from 5,000 to 3 cells, following addition of D-luciferin. (D) FACS sorting of MDA-MB-231-D3H2-shSCR and MDA-MB-231-D3H2-shEPOR cells based on tRFP status, 72 hours after 1 μg/ml doxycycline treatment. (E) Blood analysis of all mice pre-EPO administration and at the end of the experiment following twice-weekly EPO administration. RBCs, -EPO vs +EPO, $^{**}p < 0.01$; HGB, -EPO vs +EPO, $^{**}p < 0.01$; HCT, -EPO vs +EPO, $^{***}p < 0.001$; WBCs, -EPO vs +EPO, $p > 0.05$, by unpaired t-test. (F) IVIS analysis of tRFP expression in shEPOR$^{-dox}$ and shEPOR$^{+dox}$ mice.
